# RNA polymerase II coordinates histone deacetylation at active promoters

**DOI:** 10.1126/sciadv.adt3037

**Published:** 2025-02-05

**Authors:** Jackson A. Hoffman, Kevin W. Trotter, Trevor K. Archer

**Affiliations:** Epigenetics and Stem Cell Biology Laboratory, National Institute of Environmental Health Sciences, National Institutes of Health, Research Triangle Park, Durham, 27709 NC, USA.

## Abstract

Nucleosomes at promoters of active genes are marked by specific histone post-translational modifications and histone variants. These features are thought to promote the formation and maintenance of an “open” chromatin environment that is suitable for transcription. However, recent reports have drawn conflicting conclusions about whether these histone modifications depend on active transcription. To further interrogate this relationship, we inhibited transcription initiation using triptolide, which triggered degradation of RNA polymerase II, and examined the impact on histone modifications. Transcription initiation was not required for either hormone-induced or steady-state active histone modifications at transcription start sites (TSSs) and enhancers. Rather, blocking transcription initiation increased the levels of histone acetylation and H2AZ incorporation at active TSSs. P300 activity was dispensable for this effect, but inhibition of histone deacetylases masked the increased acetylation. Together, our results demonstrate that active histone modifications occur independently of transcription. Furthermore, our findings suggest that the process of transcription coordinates the removal of these modifications to limit gene activity.

## Teaser

Transcription initiation limits histone acetylation and H2AZ incorporation at promoters.

## INTRODUCTION

In transcribing the genome, the RNA polymerase II (RNAP2) complex traverses variably compacted chromatin in the form of nucleosomes and higher-order structures. Multiple protein complexes function to remodel chromatin and modify nucleosomes to permit the formation and maintenance of different genomic environments. For instance, actively transcribed regions of the genome coincide with nucleosomes containing the H2AZ histone variant and acetylated histones (*1*). However, the precise functional relationship between transcription and these active chromatin modifications is ill-defined. Recent work has concluded that several transcription start site (TSS)–associated histone modifications were dependent on transcription (*2*, *3*). Conversely, other studies have demonstrated that active marks persist upon inhibition of transcription (*4*, *5*). Moreover, inhibition of the histone acetyltransferases (HATs) and histone methyltransferases that deposit active marks can prevent recruitment of the pre-initiation complex, alter RNAP2 occupancy, and/or block transcription (*6*–*10*). Thus, despite their well-established association, the relationship between active transcription and histone modifications presents a causality dilemma (*11*).

## RESULTS

### Transcription is dispensable for steady-state and de novo H3K27 acetylation

**Fig. 1.**
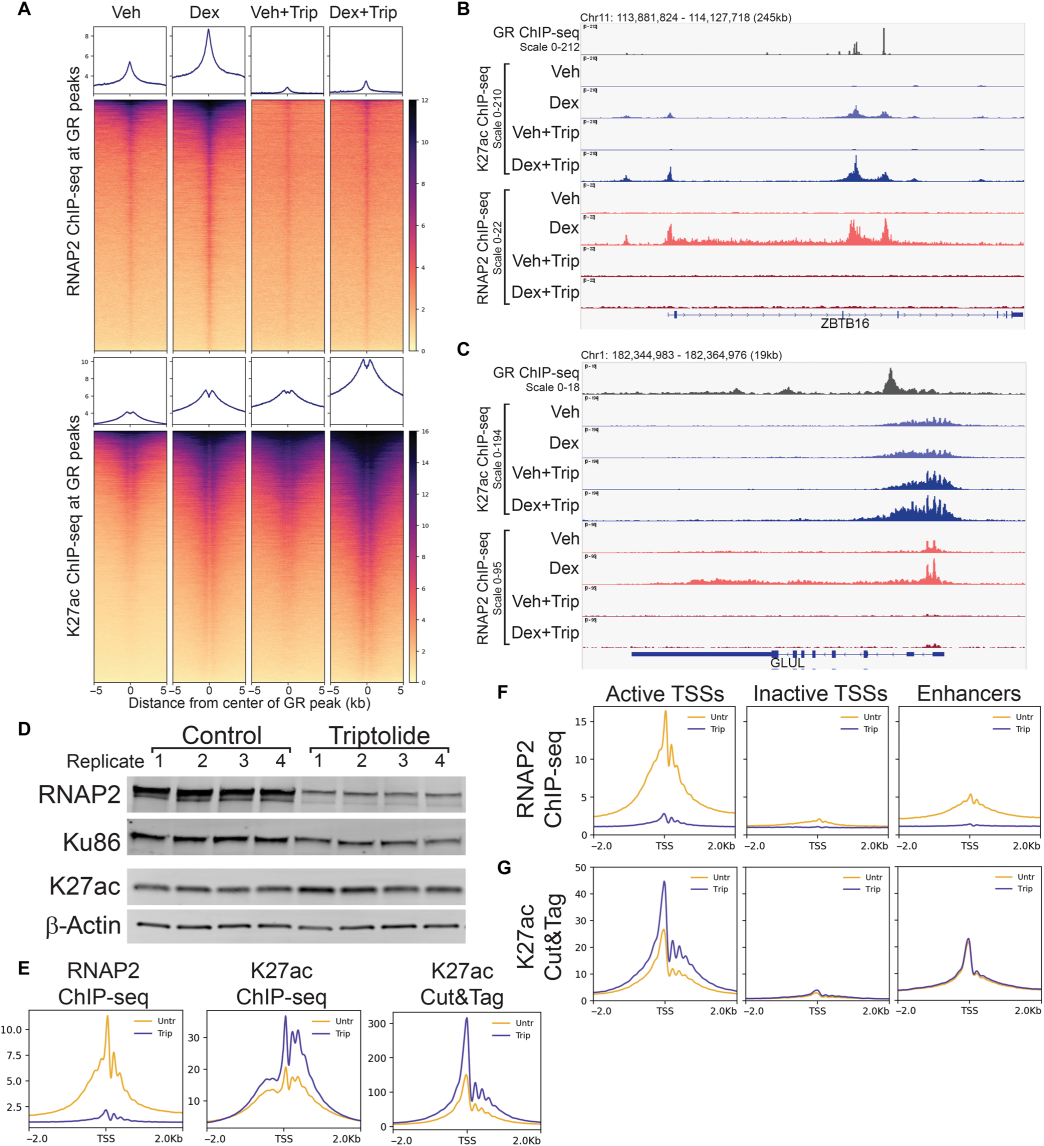
Transcription is dispensable for steady-state and de novo H3K27 acetylation. (**A**) Differential heatmap of K27ac ChIP-seq signal (log2 Dex/Veh) over class III GR peaks ± 2 hours triptolide. (**B**) Browser image of K27ac ChIP-seq and RNAP2 ChIP-seq over *ZBTB16* gene. (**C**) Browser image of K27ac ChIP-seq and RNAP2 ChIP-seq over *GLUL* gene. (**D**) Western blot of whole-cell lysates from four biological replicates each of A1-2 cells ± 2 hours triptolide. (**E**) Meta-profiles of representative ChIP-seq and Cut&Tag replicates over Refseq TSSs. (**F**) Meta-profiles of representative RNAP2 ChIP-seq ± 2 hours triptolide over Start-seq defined active, inactive, and enhancer TSSs. (**G**) Meta-profiles of representative K27ac Cut&Tag ±2 hours triptolide over Start-seq defined active, inactive, and enhancer TSSs

Stimuli-driven responses present an attractive model for resolving this dilemma, as exogenous stimuli can induce rapid changes in both transcription and the distribution of histone modifications. For instance, the synthetic glucocorticoid dexamethasone (dex) induces widespread changes in RNAP2 binding, transcription, histone acetylation, and chromatin accessibility within 1 hour of treatment in human T47D-derived A1-2 breast cancer cells (*12*). To interrogate the hierarchy of events in this response, we sought to block transcription and examine the effects on steady-state and hormone-induced histone modifications. To do so, we used triptolide, a natural product that blocks transcription initiation by inhibiting the adenosine triphosphatase activity of the XPB subunit of Transcription Factor II H (TFIIH), resulting in CDK7-dependent hyperphosphorylation and degradation of RNAP2 (*13*). We pretreated cells with triptolide for 1 hour to induce RNAP2 degradation followed by cotreatment with triptolide and dex for 1 hour. This treatment regimen resulted in degradation of RNAP2 and prevented dex-induced recruitment of RNAP2 to glucocorticoid receptor (GR) target genes such as Zinc Finger And BTB Domain Containing 16 (*ZBTB16*) and Glutamate-Ammonia Ligase (*GLUL*; Fig. 1, A to D, and fig. S1).

GR binds to ~30,000 sites in the A1-2 genome after 1 hour of dex treatment. Hormone-dependent recruitment of RNAP2 and acetylation of histone H3 lysine 27 acetylation (K27ac) is evident over these regions (Fig. 1A). While RNAP2 recruitment was mostly abolished, triptolide treatment did not prevent the hormone-induced increase in K27ac at GR peaks (Fig. 1A). Rather, K27ac levels were increased in both vehicle and dex conditions with triptolide (Fig. 1A). At *ZBTB16*, de novo, dex-dependent K27ac was observed at two intragenic GR peaks and at the TSS where GR binding was not detected (Fig. 1B). At all three sites, triptolide treatment resulted in even greater K27ac upon dex treatment (Fig. 1B). Thus, dex-dependent acetylation of K27 occurred in the absence RNAP2 recruitment and transcription initiation.

At *GLUL*, GR binding downstream of the TSS does not trigger much change in K27ac but does result in increased RNAP2 at the TSS and in the gene body (Fig. 1C). Triptolide treatment increased K27ac over the TSS independently of dex, suggesting that triptolide induced an increase in steady-state K27ac (Fig. 1C). Triptolide treatment resulted in a small increase in the overall levels of K27ac detected in whole-cell lysates and enhanced K27ac ChIP-seq signal over TSSs (Fig. 1, D and E, and fig. S1). K27ac Cut&Tag confirmed that triptolide treatment resulted in enhanced K27ac signal over TSSs (Fig. 1E).

We previously used Start-seq to identify sites of RNAP2 transcription initiation in A1-2 cells (*12*, *14*). This allowed us to classify three sets of TSSs: (i) active gene TSSs associated with Start-seq signal, (ii) nontranscribed or inactive gene TSSs, and (iii) actively transcribed enhancers. We used these TSSs subsets to determine whether the increased levels of K27ac following inhibition of initiation were restricted to TSSs where RNAP2 was active. While triptolide induced RNAP2 loss at both active TSSs and enhancers, the increased levels of K27ac were only observed at active gene TSSs (Fig. 1, F and G). This increase in K27ac levels at active gene TSSs was validated over five independent biological replicates of ChIP-seq and eight independent biological replicates of Cut&Tag (fig. S2). Thus, K27ac persisted at increased levels at active gene TSSs following inhibition of transcription initiation.

### Transcription initiation suppresses acetylation and H2AZ incorporation at active TSSs

**Fig. 2.**
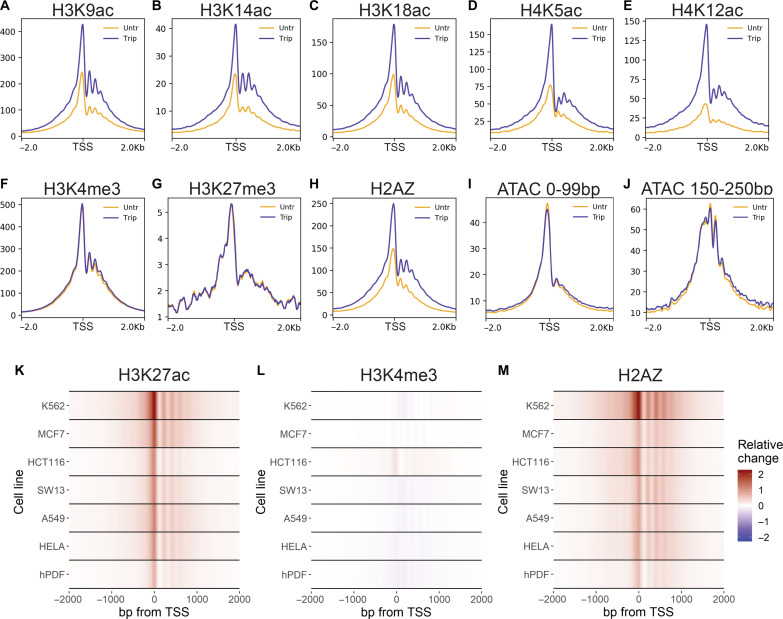
Transcription initiation suppresses acetylation and H2AZ incorporation at active TSSs. (**A** to **J**) Meta-profiles of representative Cut&Tag replicates ±2 hours triptolide over active TSSs. (**K** to **M**) Heatmaps depicting relative change [(triptolide − control)/max(control)] in Cut&Tag signal over Refseq TSSs in indicated cell lines.

To determine whether this increase was unique to K27ac, we examined additional active chromatin modifications by Cut&Tag. Inhibition of transcription initiation resulted in increased levels of acetylation at H3K9, K3K14, H3K18, H4K5, and H4K12 at active TSSs without noticeable change in bulk levels (Fig. 2, A to E, and fig. S3). However, tri-methylation of H3K4 (H3K4me3) at active TSSs was unaffected by inhibition of initiation (Fig. 2F and fig. S3). Similarly, the H3K27me3 repressive mark was also unaffected (Fig. 2G). Thus, both acetylation and methylation of histones at TSSs persisted, and the levels of histone acetylation were increased in the absence of transcription initiation.

Active TSSs are also marked by the replacement of histone H2A with the histone variant H2AZ. We also observed enhanced levels of the H2AZ incorporation at active TSSs after inhibition of transcription initiation (Fig. 2H). Although H3K4me3 was unchanged, the observed increase in acetylation and H2AZ incorporation could be caused by a change in the nucleosome occupancy following triptolide treatment. To test this, we performed assay for transposase-accessible chromatin using sequencing (ATAC-seq) to examine chromatin accessibility at active TSSs. Inhibition of initiation had little effect on the profile of either short, nucleosome-free fragments or nucleosome-size fragments (Fig. 2, I and J). Therefore, the inhibition of transcription initiation enhanced both histone acetylation and H2AZ incorporation on nucleosomes at active TSSs.

To further validate these findings, we examined the effect of transcription inhibition on K27ac, H3K4me3, and H2AZ enrichment in additional cell lines. As in A1-2 cells, we observed increased K27ac and H2AZ at TSSs following triptolide treatment in human lymphoma (K562), breast (MCF7), colon (HCT116), adrenal gland (SW13), lung (A549), and cervical (HeLa) cancer cell lines and in primary dermal fibroblasts (Fig. 2, K and M). H3K4me3 remained largely unchanged after triptolide treatment in each of these cell lines, apart from HCT116 cells, which exhibited a marginally increased level (Fig. 2L). Thus, the effects of inhibiting transcription initiation were highly reproducible and largely independent of cell line and tissue of origin.

Previously, inhibition of transcription with triptolide has been shown to trigger a global loss of K27ac and H3K4me3 in K562 cells (*3*). The chromatin immunoprecipitation sequencing (ChIP-seq) experiments performed in this study followed a different protocol with milder cross-linking and micrococcal nuclease (MNase) fragmentation (*3*). To determine whether the discrepancy in findings was due to technical differences, we repeated our ChIP-seq experiment using these conditions and reagents. With this alternative protocol, we observed similar profiles of increased K27ac and H2AZ along with unchanged H3K4me3 at TSSs following triptolide treatment (fig. S4). Hence, our findings are consistent across distinct ChIP-seq protocols and Cut&Tag.

As triptolide rapidly blocks transcription initiation, we sought to profile the changes in acetylation and H2AZ incorporation over a time course of treatment. Increased levels of K27ac and H2AZ were observed within 15 min of triptolide treatment, with a further increase at 30 min (fig. S5). Together, these experiments demonstrated that blocking transcription initiation rapidly resulted in enhanced acetylation and H2AZ incorporation at TSSs.

### RNAP2 degradation reproduces the effect of inhibiting transcription inhibition

**Fig. 3.**
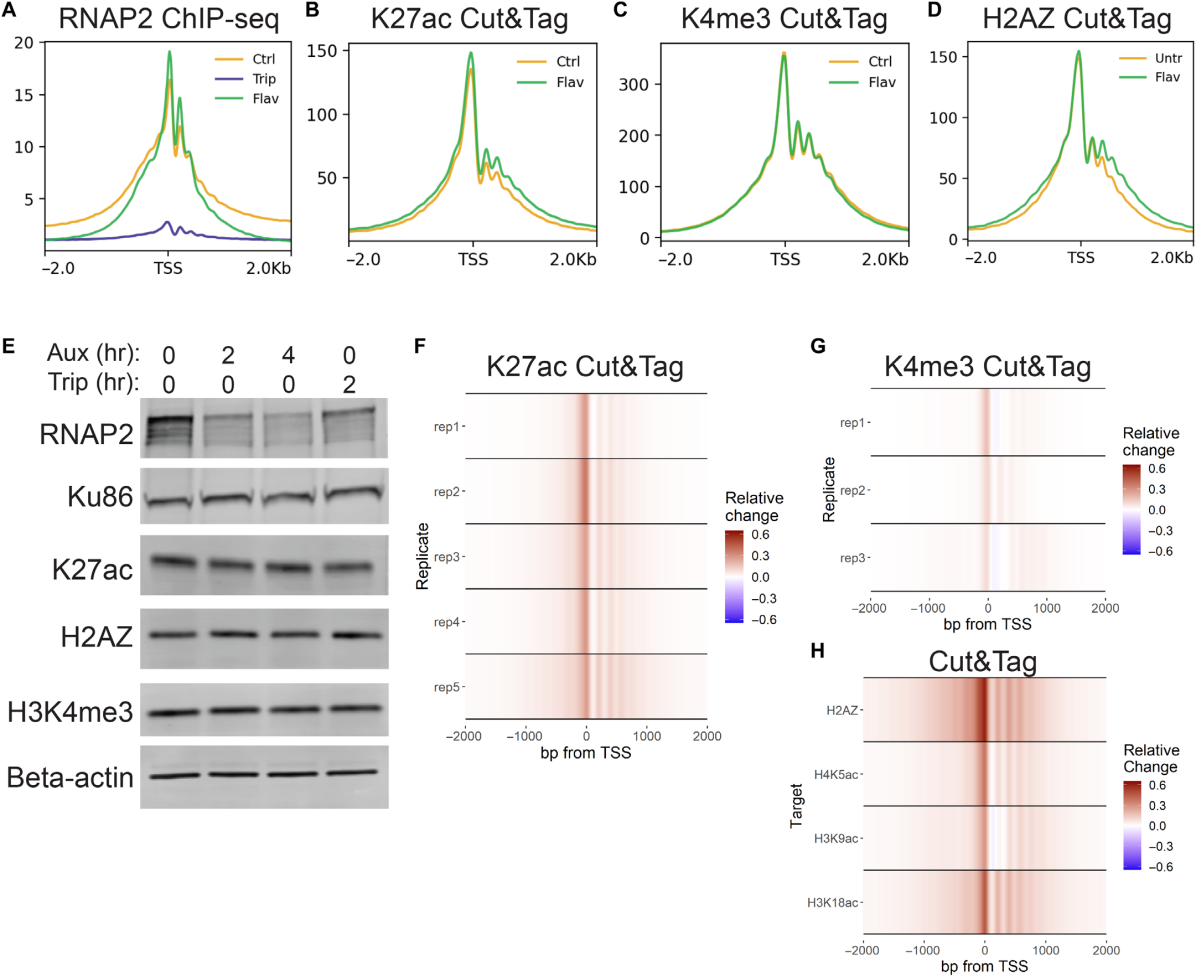
RNAP2 degradation reproduces the effect of inhibiting transcription inhibition. (**A** to **D**) Meta-profiles of ChIP-seq and Cut&Tag signal ±2 hours flavopiridol over active TSSs. (**E**) Western blots of whole-cell lysate from HCT116-mAID-*POLR2A* cells ± auxin or triptolide. (**F** to **H**) Heatmaps depicting relative change [(auxin − control)/max(control)] in Cut&Tag signal for indicated marks.

To investigate whether the enhanced acetylation and H2AZ incorporation was caused by the loss of transcription initiation or RNAP2 degradation, we used an alternative method of inhibiting transcription. Flavopiridol inhibits CDK9 and prevents phosphorylation of Negative Elongation Factor (NELF) and DRB Sensitivity Inducing Factor (DSIF) to block pause release (*15*). Treatment with flavopiridol for 2 hours stalled RNAP2 at TSSs and blocked transcription elongation (Fig. 3A). This resulted in a marginal increase in K27ac, unchanged H3K4me3, and unchanged H2AZ at active TSSs (Fig. 3, B to D). Thus, blocking transcription by inhibiting pause release was not sufficient to trigger the increased histone acetylation observed after blocking transcription initiation.

We next sought to determine whether RNAP2 degradation was sufficient to replicate the effect of triptolide on histone acetylation and H2AZ incorporation. To do so, we used an auxin-inducible degron (AID) system in HCT116 cells in which RNA Polymerase II Subunit B1 (RPB1) is bi-allelically tagged with a mini-AID and mClover cassette (*16*). These cells also harbor a doxycycline-inducible cassette for expression of the *Oryza sativa* auxin-binding protein TIR1 (osTIR). Following 48 hours of doxycycline induction of osTIR expression, rapid degradation of RNAP2 can be triggered by treatment with auxin. Two hours of auxin treatment was sufficient to reduce RNAP2 similarly to 2 hours of triptolide treatment without noticeable change in the bulk levels of histone modifications (Fig. 3E and fig. S6). This degradation of RNAP2 resulted in reproducibly increased K27ac at active TSSs, albeit a lower level than observed with triptolide (Fig. 3F). A milder increase was observed in H3K4me3, similar to that caused by triptolide in HCT116 cells (Fig. 3G). Levels of H2AZ incorporation, H3K9ac, H3K18ac, and H4K5ac were also increased after AID degradation of RNAP2 (Fig. 3H). This revealed that depletion of RNAP2 without inhibition of transcriptional activity could also trigger enhanced histone acetylation and H2AZ incorporation at TSSs.

### HDAC inhibition masks the effect of inhibiting transcription initiation

**Fig. 4.**
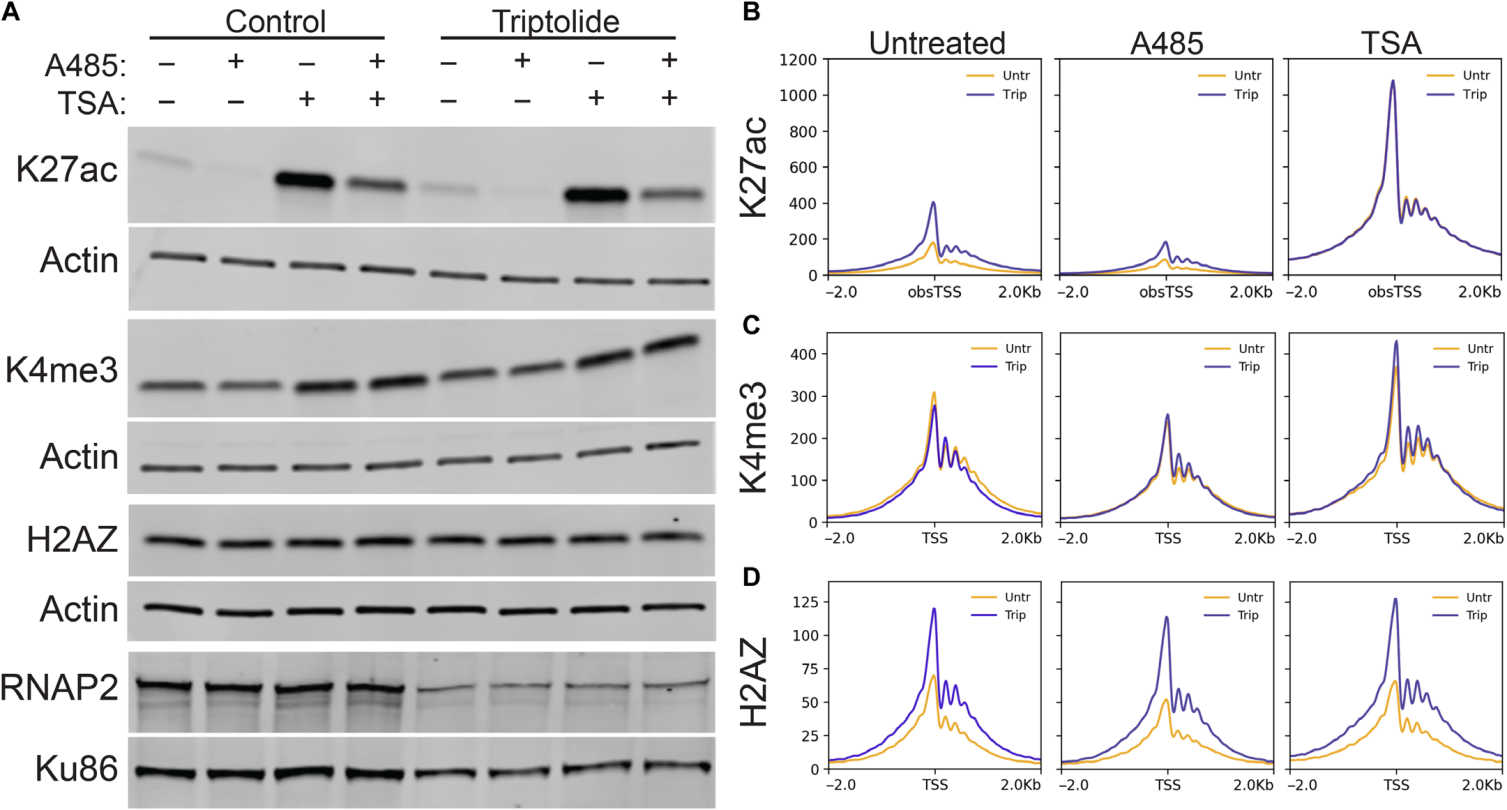
HDAC inhibition masks the effect of inhibiting transcription initiation. (**A**) Western blots of whole-cell lysates from A1-2 cells treated ± triptolide, A485, and TSA. (**B** to **D**) Meta-profiles of Cut&Tag signal in A1-2 cells treated ± triptolide, A485, and TSA.

Histone acetylation is dynamically regulated by the activity of HATs and histone deacetylases (HDACs), which respectively catalyze the deposition and removal of acetyl marks. We next sought to determine whether the increased acetylation caused by blocking transcription initiation was the result of increased HAT activity or a loss of HDAC activity. To test HAT dependence, we used the P300 inhibitor A485 (*17*). P300 is the predominant HAT responsible for K27ac at TSSs (*18*). Treatment with A485 diminished the levels of K27ac detected in cell lysates and at TSSs but had little effect on H3K4me3 or H2AZ (Fig. 4, A to D, and fig. S7). Despite the reduced overall levels of K27ac, A485 did not prevent the triptolide-induced increase in K27ac or H2AZ at active TSSs. Thus, the gained acetylation at active TSSs was not dependent on P300 activity.

To test whether a loss of HDAC activity was responsible for the gained acetylation, we treated cells with trichostatin A (TSA), an inhibitor of class I and class II HDACs (*19*). TSA treatment markedly increased the levels of K27ac in cell lysates and at active TSSs (Fig. 4, A and B, and fig. S7). Unlike A485, no difference was observed in the level of K27ac at active TSSs following inhibition of transcription initiation in the presence of TSA (Fig. 4B). This suggested that the increase in K27ac at active TSSs was caused by a loss of HDAC activity following inhibition of transcription initiation.

TSA had no effect on the overall level of H2AZ but did result in an overall increase in H3K4me3 (Fig. 4, A, C, and D, and fig. S7). However, H3K4me3 levels remained unaffected by triptolide in TSA-treated cells (Fig. 4C). Conversely, inhibition of transcription initiation still enhanced H2AZ incorporation after TSA treatment (Fig. 4D). Together, this indicated that histone acetylation and H2AZ incorporation were independently regulated at active TSSs and both processes were enhanced by inhibition of transcription initiation.

### The relationship between transcription and histone modifications

In this study, we show that inhibition of transcription initiation causes an increase in histone acetylation and H2AZ incorporation at active TSSs. Furthermore, blocking transcription initiation does not prevent de novo histone acetylation at hormone induced TSSs and enhancers. We thus conclude that the placement of these active chromatin marks is not specifically dependent on transcription.

These findings contrast with previous reports that concluded that active histone modifications are a result of active transcription (*2*, *3*). We reasoned that technical differences in chromatin preparation for ChIP-seq could be responsible these discrepancies. Nucleosomes surrounding active TSSs are characterized by their fragility during enzymatic chromatin fragmentations (*20*). These fragile nucleosomes are likely the most enriched for acetylation and H2AZ incorporation following triptolide treatment. Hence, loss of these nucleosomes as a result of insufficient cross-linking or overdigestion/fragmentation of the chromatin would be more pronounced in triptolide-treated cells and lead to the appearance of lower levels of TSS-associated histone modifications. We validated and expanded on our ChIP-seq findings with Cut&Tag, which uses intact nuclei and should not be affected by nucleosome fragility.

While inhibition of p300/CBP HAT activity reduced the overall levels of acetylation at TSSs, it did not prevent the triptolide-induced increase in acetylation. This indicated that the gained acetylation at TSSs was not dependent on the deposition of new acetylation by HATs. Conversely, HDAC inhibition increased the overall levels of TSS acetylation, and no further increased in histone acetylation was observed in combined TSA and triptolide treatment. As the gained acetylation was fully masked by HDAC inhibition, this suggested that inhibition of transcription initiation also resulted in a loss of HDAC activity at active TSSs.

H2AZ incorporation occurred independently of both transcription and histone acetylation, as neither the overall H2AZ levels nor the effect of triptolide on H2AZ incorporation was altered by HAT or HDAC inhibition. Thus, inhibition of transcription initiation was independently associated with both a loss of HDAC activity and the retention of H2AZ. Our findings suggest that their activity is recruited or promoted by the initiation of transcription. HDACs and the TIP60/P400, SRCAP, and INO80 complexes that regulate H2AZ incorporation indeed localize to active TSSs and are recruited to hormone-induced promoters during initiation (*21*–*25*). Intriguingly, live imaging studies have shown that acetylation precedes initiation and that initiated RNAP2 and K27ac-modified histones occupy separate chromatin regions (*26*, *27*). These observations support a model in which the presence of acetylation and H2AZ poises TSSs for initiation, upon which these marks are actively removed from the chromatin.

Auxin-inducible degradation of RNAP2 alone reproduced the effect of triptolide on acetylation and H2AZ levels at TSSs. However, while similar reduction of RPB1 levels were achieved with 2 hours of auxin or triptolide in HCT116-mAID-*POLR2A* cells, degradation alone yielded a smaller increase in acetylation and H2AZ levels than observed following triptolide treatment. As the remaining RNAP2 in auxin-treated cells may still initiate transcription, this suggests that the process of transcription initiation recruits or facilitates the activity of HDACs and the complexes that govern H2AZ incorporation at active TSSs. RNAP2 unwraps DNA from nucleosomes and generates hexasomes, providing more enzymatically favorable substrates for HDACs and H2AZ remodelers (*10*, *28*–*30*).

At active TSSs, histone deacetylation and H2AZ removal are transcriptionally repressive or inhibitory changes to the chromatin landscape. Hence, their association with transcription initiation appears counterintuitive. However, these changes may represent a transcriptionally coupled mechanism to govern gene activity. Histone acetylation influences transcriptional bursting dynamics, which can be measured by how often an individual allele is actively transcribed (burst frequency) and/or the number of mRNA produced when an allele is activated (burst size). Targeted approaches using CrisprA demonstrate that directing acetylation to specific promoters increases the size and frequency of transcriptional bursts, and global inhibition of HDACs also leads to increases in burst size (*31*). Thus, transcription associated HDAC activity may function in the native context to limit bursting dynamics.

In contrast to the changes in histone acetylation and H2AZ incorporation, the levels of H3K4me3 were unaffected by inhibition of transcription initiation. Furthermore, inhibition of HAT activity did not alter the levels of H3K4me3. This supports findings that suggest that H3K4me3 precedes K27ac in the hierarchy of TSS modifications and functions a bookmark for active TSSs (*32*). Minute-by-minute ChIP analysis at an estrogen-inducible promoter revealed that the H3K4me3 histone methyltransferase PRMT1 was recruited before P300 (*25*). In addition, de novo induction of H3K4me3 by CrisprA induces K27ac (*33*). Furthermore, while H3K4me3 and K27ac can both induce transcriptional activation when installed de novo at a promoter, the effect of de novo H3K4me3 was markedly reduced by inhibition of HAT activity (*33*). Therefore, our findings support a model suggesting that H3K4me3 marks TSSs that can be activated, and subsequent histone acetylation promotes initiation of transcription.

Unexpectedly, the effect of blocking transcription initiation on histone acetylation levels was limited to active TSSs and did not have a robust effect on histone acetylation at active enhancers. RNAP2 and general transcription factors including TFIID and TFIIH are recruited to both TSSs and enhancers (*34*). This suggests that an underlying difference between TSSs and enhancers alters the ability of RNAP2 to recruit or direct the activity of histone modifying proteins. The methylation status of H3K4 may play a role in this difference. While mono-, di-, and tri-methylation of H3K4 are observed at both enhancers and TSSs, the ratio of H3K4me1:H3K4me3 is increased at enhancers (*35*). However, the differences between enhancers and TSSs are not definitive, and it can be difficult to distinguish enhancers and TSSs based solely on their chromatin landscapes. Further examination of the determinants governing the effect of transcription on histone modifications at TSSs may provide greater distinction between TSSs and enhancers.

If the role of histone acetylation at TSSs is to promote bursting and/or initiation of transcription, then genome-wide hyperacetylation of TSSs would likely cause global alterations in the cellular transcriptional profile. The hyperacetylation observed after triptolide treatment likely represents a greater proportion of alleles with acetylated TSSs. After recovering from triptolide treatment, RNAP2 is the probable rate-limiting factor in the reestablishment of the transcriptome. As RNAP2 levels recover, the increased number of acetylated and initiation competent TSSs may result in a flattening of the global transcriptional profile, with enhanced expression of low burst size genes and dampened expression of high burst size genes. Several studies have demonstrated that HDAC inhibition has complex effects on gene expression and often broadly disrupts the transcriptome. A striking example of this was observed during zygotic genome activation (ZGA) in mouse embryos, the process during which transcription first occurs in the embryonic genome. HDAC inhibition before ZGA resulted in global alteration of the transcriptome once ZGA began, with statistically significant changes in expression observed at ~3000 genes (*36*). In addition, inhibition of P300/CBP HAT activity greatly disrupted the transcriptome during post-mitotic genome activation, indicating a requirement for histone acetylation in reestablishing the transcriptome following mitosis (*32*). Hence, histone acetylation levels appear to be both determined by and a determinant of active transcription.

## MATERIALS AND METHODS

### Cell lines and treatments

Human T47D A1-2 (*37*) and MCF-7 cell were grown in minimum essential medium (MEM; Gibco, 10370) supplemented with 10% fetal bovine serum (Gibco, 26140-079), GlutaMAX (Gibco, 3505), Hepes (Sigma-Aldrich, H0887), and penicillin/streptomycin (Sigma-Aldrich, P0781). HeLa, SW-13, U2OS, primary human fibroblast, and mouse C127 cells were grown in Dulbecco’s MEM (Gibco, 11965-092) supplemented with 10% fetal bovine serum (Gibco, 26140-079), GlutaMAX (Gibco, 3505), Hepes (Sigma-Aldrich, H0887), and penicillin/streptomycin (Sigma-Aldrich, P0781). K562 cells were grown in suspension in RPMI Medium 1640 (Gibco, 11875-093) supplemented with 10% fetal bovine serum (Gibco, 26140-079), GlutaMAX (Gibco, 3505), Hepes (Sigma-Aldrich, H0887), and penicillin/streptomycin (Sigma-Aldrich, P0781).

All cells were cultured in a humidified incubator at 37°C with 5% CO_2_ in the indicated growth medium with media changed every 2 to 3 days. Cells were seeded in new tissue culture treated dishes when 80% confluency reached.

Cells were treated for the indicated time individually, or in combination, with 100 nM dex (Sigma-Aldrich, D4902), 0.5 μM triptolide (Tocris, 3253), 0.5 μM flavopiridol (Tocris, 3094), 0.5 μM A485 (Tocris, 6387), 0.1 μM TSA (Sigma-Aldrich, T8552), doxycycline (10 μg/ml; Sigma-Aldrich, D9891), 1 μM 5-Ph-IAA (AID) ligand (Sigma-Aldrich, SML3574), or vehicle (equal volume EtOH or dimethyl sulfoxide).

### Immunoblot analysis

Whole-cell and nuclear protein extracts were prepared from cell or nuclei aliquots taken after indicated treatment and before downstream assay analysis. Compact cell or nuclei pellets were resuspended in RIPA-HS buffer [25 mM tris HCl (pH 7.6), 500 mM NaCl, 1% NP-40, 1% sodium deoxycholate, 0.1% SDS, 100 μM phenylmethylsulfonyl fluoride (PMSF), and 1× cOmplete EDTA-free protease inhibitors (Roche)], incubated on ice for 15 min, then subjected to sonication using PIXUL Multi-Sample Sonicator (Active Motif) at 12°C using the following parameters: Pulse [N]: 50; PRF [kHz]: 1.00; Process Time: 60 sec; Burst Rate [Hz]: 20.00. After sonication, samples were centrifugated at 14,000*g* for 15 min and protein extract collected as supernatant. Protein concentration determined by Bradford assay. Whole cell (50 μg) or nuclear (5 μg) were resolved using SDS–polyacrylamide gel electrophoresis, transferred to low-fluorescence polyvinylidene difluoride membrane (Bio-rad), and blocked in TBS blocking buffer (Li-Cor). Immunoblot analysis was performed as previously described (*38*) using the indicated antibodies as described in table S1 with a direct near-infrared fluorescence detection system (Li-cor Odyssey CLx Imaging System).

### ChIP-seq

Cells were fixed in phosphate-buffered saline (PBS) with 1% methanol-free formaldehyde (Thermo Fisher Scientific, 28906) at 37°C for 10 min and quenched with glycine (0.125 M) for 5 min at room temperature. After glycine quench, cells were washed three times and scraped into ice-cold PBS and pelleted by centrifugations at 500*g* for 5 min. Compact cell pellets were resuspended in MNase swelling buffer [25 mM Hepes (pH 7.9), 1.5 mM MgCl_2_, 10 mM KCl, 0.1% NP-40, 0.5 mM PMSF, and 1× cOmplete EDTA-free protease inhibitors (PIC)] (Roche), incubated on ice for 10 min then subjected to Dounce homogenization using 20 strokes with “tight” pestle (Duran Wheaton Kimble, 357542). Nuclei were sedimented through digestion buffer [15 mM Hepes (pH 7.9), 60 mM KCl, 15 mM NaCl, 0.32 M sucrose, 0.5 mM PMSF, and PIC] by centrifugation for 930*g* for 7 min at 4°C. Nuclei pellets were fully resuspended in digestion buffer containing 3.3 mM CaCl_2_ and digested with MNase (0.75 U/1 × 10^6^ cells, Worthington) at 37°C for 15 min. MNase fragmentation was stopped by addition of 10 mM EGTA and incubation for 5 min on ice. Digested nuclei were centrifuged at 930*g* for 7 min at 4°C and compact nuclei pellets resuspended in 20 volumes (v/v) ChIP Shearing buffer [50 mM Hepes pH 7.9, 140 mM NaCl, 1 mM EDTA, 1% Triton X-100, 0.1% Na-deoxycholate, 0.1% SDS containing spermidine, PIC, and PMSF] and incubated on ice for 10 min. Samples were subjected to sonication using PIXUL Multi-Sample Sonicator (Active Motif) at 12°C using the following parameters: Pulse [N]: 50; PRF [kHz]: 1.00; Process Time: 10 min; Burst Rate [Hz]: 20.00. After sonication, fragmented chromatin was recovered in supernatant after centrifugation at 18,000*g* for 10 min with extent of fragmentation determined by agarose gel electrophorese before immunoprecipitation. The concentration of fragmented chromatin was determined by Bradford assay. Fragmented chromatin was diluted in twofold in 2× IP buffer [20 mM tris-HCl (pH 8.0), 300 mM NaCl, 2 mM EDTA, 20% glycerol, 1% Triton X-100, 0.5 mM PMSF, and PIC] and immunoprecipitation performed with the indicated antibodies at a ratio of 1-μg antibody per 200-μg chromatin. Equal amounts of Spike-IN antibody and *Drosophila* chromatin (Active Motif, 61686 and 53083) were added to each immunoprecipitation for normalization. Immune complexes were captured using protein G dynabeads (Invitrogen, 10004D), washed once each with low-salt buffer [20 mM tris-HCl (pH 8.0), 150 mM NaCl, 2 mM EDTA, 1% Triton X-100, and 0.1% SDS], high-salt buffer (same as low-salt buffer, except 500 mM NaCl), and LiCl buffer [tris-HCl (pH 8.0), 250 mM LiCl, 2 mM EDTA, 1% NP-40, and 1% (w/v) sodium deoxycholate], and twice with TE. Immunoprecipitated DNA was recovered from beads by incubation in ChIP elution buffer (1% SDS and 0.1 M sodium bicarbonate) for 30 min at room temperature. Eluted DNA was treated with RNase A (Roche, 10109169001) and proteinase K (Invitrogen, #25530015) and purified using Qiagen polymerase chain reaction (PCR) purification columns.

ChIP-seq in K562 cells with milder cross-linking and fragmentation performed using a SimpleChIP Plus Enzymatic Chromatin IP Kit with Magnetic Beads (Cell Signaling Technologies #9005S) according to manufacturer’s protocol. ChIP-seq libraries were generated using the xGen ssDNA & Low-Input DNA Library Prep Kit and xGen CDI Primers (IDT, 10009817 and 10009815) according to manufacturer’s protocol and sequenced on the Illumina NextSeq platforms.

### ChIP-seq analysis

Reads were trimmed using Cutadapt (*39*) and aligned to both hg19 and dm6 genomes using Bowtie2 (*40*). Aligned reads were deduplicated with picardtools (http://broadinstitute.github.io/picard). Scale factors were calculated for each sample by dividing the minimum number of dm6 reads for a given antibody by number of dm6 reads for each sample using the same antibody. Scaled and normalized coverage files were generated using deepTools (*41*) with calculated scale factors and rpkm normalization. Meta-profiles and heatmaps were generated using deepTools and ggplot2.

### Cut&Tag

Cells were treated, harvested, and subjected to Cut&Tag analysis using a modified published protocol (*42*). Trypsinized cells were washed once with PBS and incubated with NE nuclear extraction buffer [20 mM Hepes-KOH (pH 7.9), 10 mM KCl, 0.1% Triton X-100, 20% glycerol, 0.5 mM spermidine, 100 μM PMSF, and 1× EDTA-free protease inhibitors (Roche cOmplete)] for 30 min on ice followed by Dounce homogenization using 20 strokes from Dounce Tissue Grinder (Wheaton 357542) tight pestle. Following homogenization, samples were centrifuged for 3 min at 600*g*, nuclei pellet resuspended in NE buffer supplemented with 0.1% formaldehyde, and incubated at room temperature for 2 min followed by quench with 0.125 M glycine. Following formaldehyde cross-link, nuclei were recentrifuged, resuspended in fresh NE buffer, and counted with trypan blue dye using Bio-Rad TC20 Automated Cell Counter.

After isolation, 150,000 total nuclei (135,000 nuclei from experimental sample with 15,000 mouse spike-in nuclei) were incubated with activated concanavalin A–coated (ConA) magnetic beads (EpiCypher) in 0.2-ml thin-walled tubes for 10 min at room temperature. Nuclei-bound ConA beads were resuspended in antibody incubation buffer [20 mM Hepes (pH 7.5), 150 mM NaCl, 0.01% digitonin, 2 mM EDTA, 0.5 mM spermidine, 100 μM PMSF, and 1× EDTA-free protease inhibitor], 0.75 μg of indicated primary antibody (see table S2) added, and samples incubated overnight at 4°C with gentle rotation. Primary antibody removed from ConA bead solution by placing samples on magnetic stand until clear followed by supernatant removal. Samples were incubated with 0.75 μg of species-specific secondary antibody for 30 min at room temperature with gentle rotation followed by two washes with Digitonin150 wash buffer [20 mM Hepes (pH 7.5), 150 mM NaCl, 0.01% digitonin, 0.5 mM spermidine, 100 μM PMSF, and 1× EDTA-free protease inhibitor] then incubation with pAG-Tn5 in Digitonin300-wash buffer [20 mM Hepes (pH 7.5), 300 mM NaCl, 0.01% digitonin, 0.5 mM spermidine, 100 μM PMSF, and 1× EDTA-free protease inhibitor] for 1 hour at room temperature.

After pAG-Tn5 incubation, bound ConA beads were washed twice with Digitonin300-wash buffer then resuspended in Tagmentation buffer (Digitonin300 wash buffer containing 10 mM MgCl_2_) and incubated for 1 hour at 37°C. Beads were then pelleted and resuspended in TAPS buffer [10 mM TAPS (pH 8.5) and 0.2 mM EDTA]. TAPS buffer was removed and beads were carefully resuspended in SDS release buffer (10 mM TAPS pH 8.5, 0.1% SDS) and incubated for 1 hour at 58°C. Following incubation, SDS quench buffer (0.67% Triton X-100) was added to each sample along with 2 μl of a universal i5 and a uniquely barcoded i7 primers (from 10 μM stocks). Equal volume (25 μl) of NEBNext High-Fidelity 2× PCR Master mix (M0541, New England Biolabs) was added to each sample and mixed by gentle pipetting. Samples subjected to DNA amplification using a thermocycler and the CUT&TAG-specific PCR cycling parameters: 58°C for 5 min; 72°C for 5 min; 98°C for 45 s; with 19 cycles of 98°C for 15 s and 60°C for 10 s; final extension at 72°C for 1 min; and hold at 4°C. DNA cleanup was performed by adding 1.3× AMPure XP beads (A63880, Beckman Coulter) to each sample, allowing libraries to incubated with beads for 10 min at room temperature, followed by two washes with 80% ethanol, then elution in 15 μl of 10 mM tris (pH 8.0). While on magnetic stand, supernatant was carefully taken and transferred to a fresh tube. Library size distribution determined by Agilent TapeStation 4200 (Agilent Technologies) and libraries mixed to yield equal representation before paired-end (2 × 50 bp) Illumina sequencing by NIEHS The Epigenomics and DNA Sequencing Core Facility.

### Cut&Tag analysis

Reads were trimmed using Cutadapt (*39*) and aligned to both hg19 and mm10 genomes using Bowtie2 (*40*). Aligned reads were deduplicated with picardtools. Deduplicated read counts were used to calculate the human:mouse ratio, which was then used to calculate scaling factors for samples performed side by side with the same antibody. The scale factor was calculated by dividing the human:mouse read ratio for each sample using a given antibody by the maximum human:mouse read ratio from samples using the same antibody. Scaled and normalized coverage files were generated using deepTools (*41*) with calculated scale factors and rpkm normalization. Meta-profiles and heatmaps were generated using deepTools and ggplot2.

### ATAC-seq

Two replicates were used for each treatment condition. Isolated cells were treated in 10 mM PIPES (pH 6.8), 100 mM NaCl, 300 mM sucrose, 3 mM MgCl_2_, and 0.1% Triton X-100 and then treated with the Illumina Tagment DNA TDE1 Enzyme and Buffer Kits for 30 min followed by mixing every 10 min. Isolation of libraries and amplification was performed as described (*43*). Libraries were sequenced at the NIEHS Epigenomics Core Facility on a NovaSeq instrument, and reads were trimmed with default parameters.
