# Supplementary material for: RNA polymerase II coordinates histone deacetylation at active promoters
Source: Sci Adv. 2025 Feb 5;11(6):eadt3037. doi: 10.1126/sciadv.adt3037 (PMC11797538; doi:10.1126/sciadv.adt3037)
Supplement: Supplementary file 1 — Figs. S1 to S7 Tables S1 and S2 [file sciadv.adt3037_sm.pdf]

Science Advances
AAAS

Supplementary Materials for

RNA polymerase II coordinates histone deacetylation at active promoters

Jackson A. Hoffman *et al.*

Corresponding author: Jackson A. Hoffman, jackson.hoffman@nih.gov; Trevor K. Archer, archer1@niehs.nih.gov

Sci. Adv. **11**, eadt3037 (2025)
DOI: 10.1126/sciadv.adt3037

This PDF file includes:

Figs. S1 to S7
Tables S1 and S2

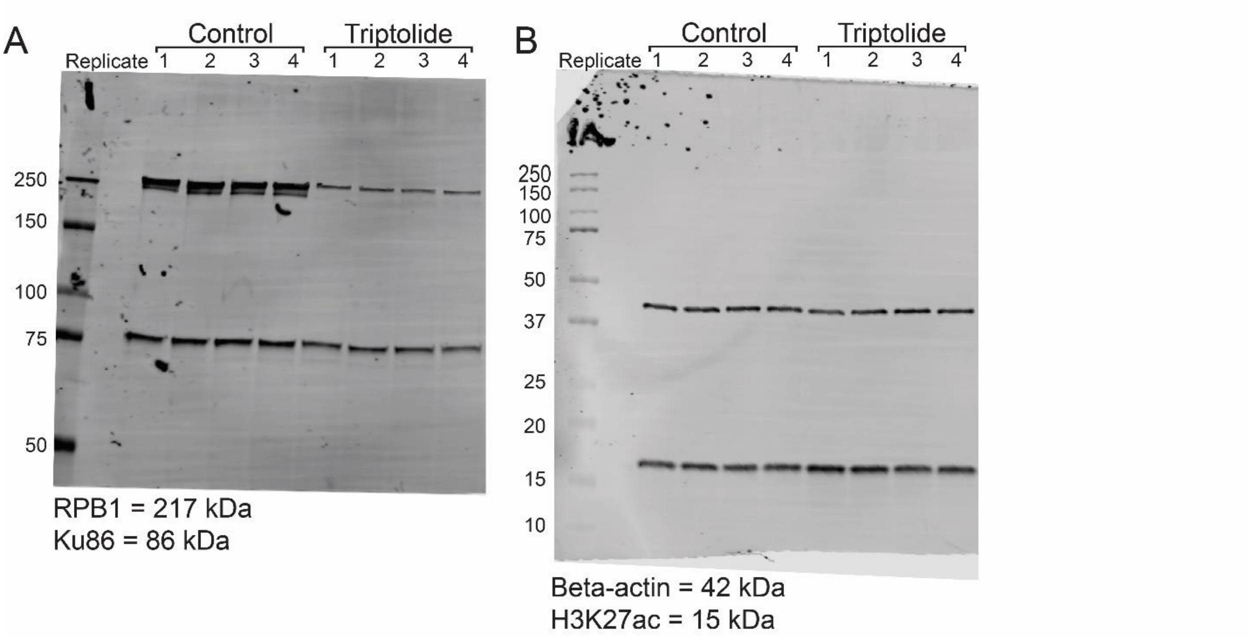

Figure S1: Uncropped western blot images corresponding to Figure 1D.

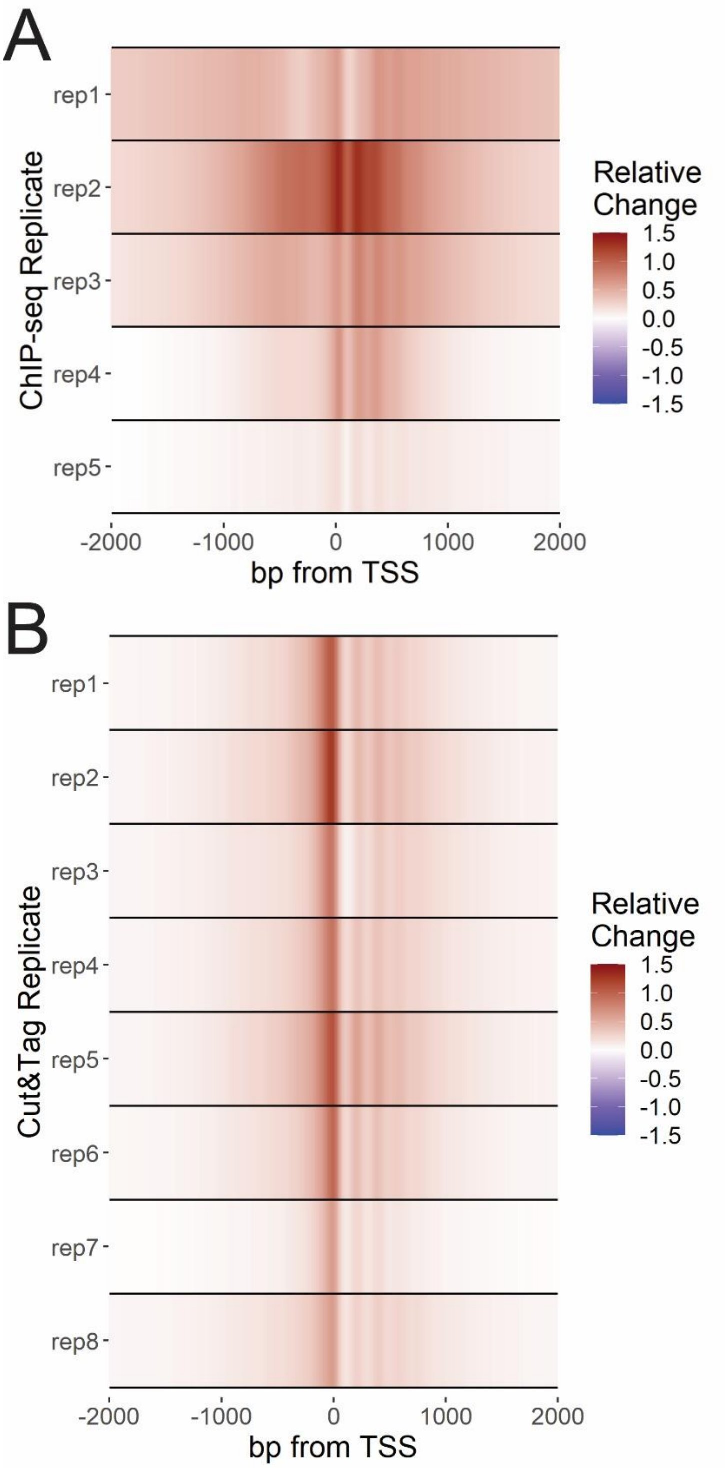

Figure S2: A) Heatmaps depicting relative change ((Triptolide – control) / max(control)) in K27ac ChIP-seq signal across 5 independent biological replicates. B) Heatmaps depicting relative change ((Triptolide – control) / max(control)) in K27ac Cut&Tag signal across 8 independent biological replicates.

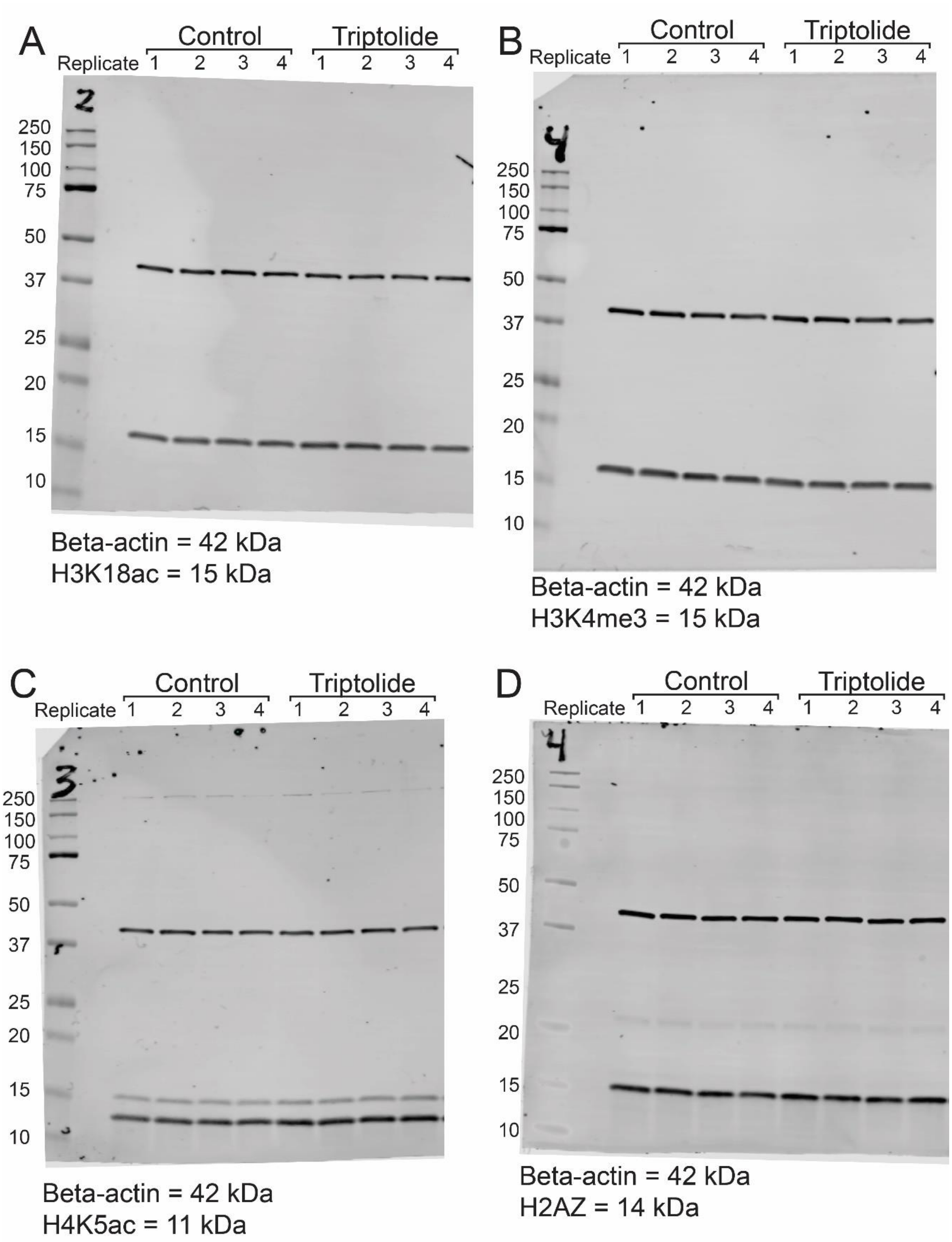

Figure S3: Uncropped western blot images corresponding to Figure 2.

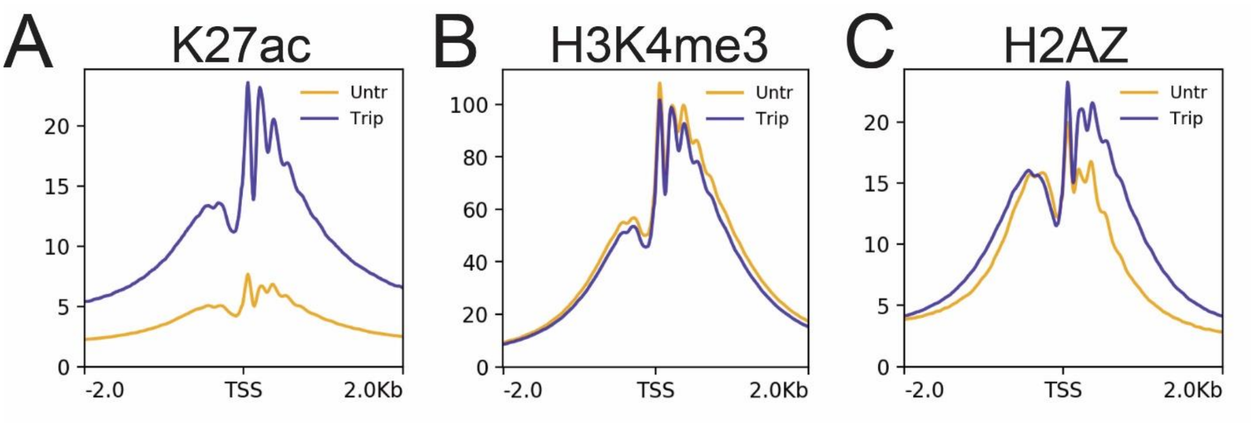

Figure S4: Meta-profiles of ChIP-seq signal +/- 2 hours triptolide over Refseq TSSs in K562 cells processed with mild fixation and MNase fragmentation protocol.

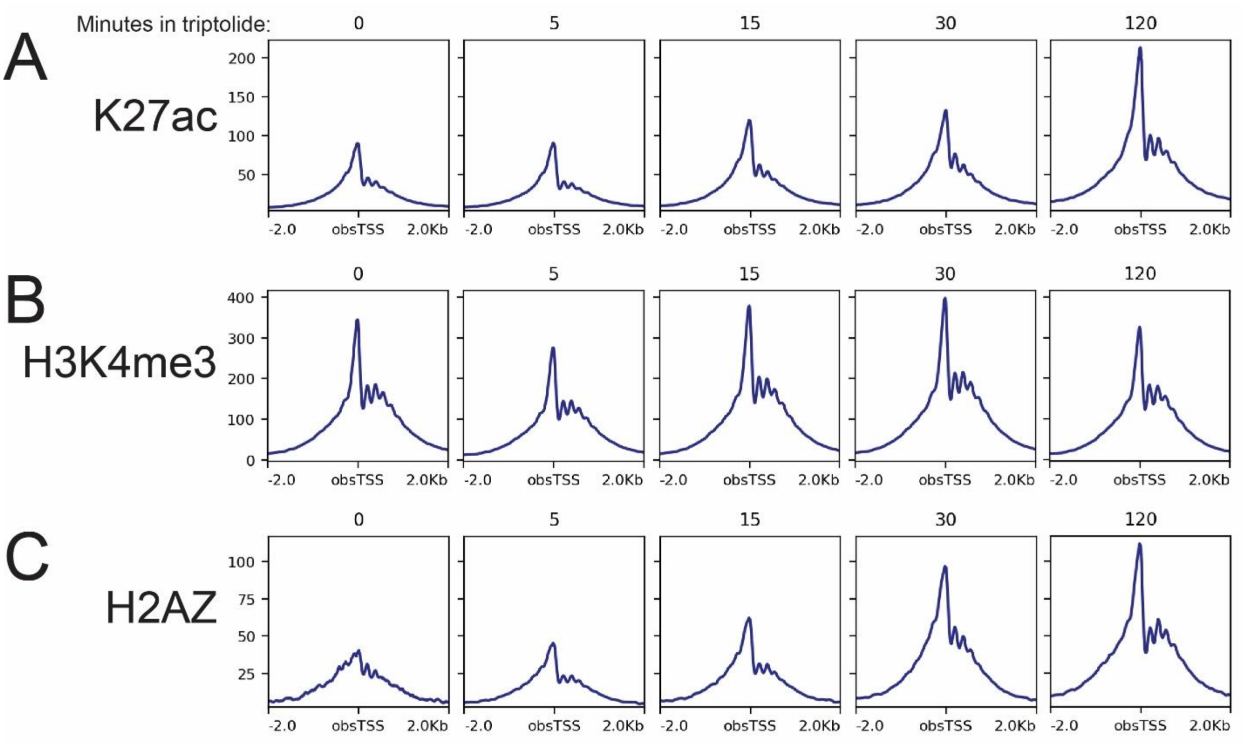

Figure S5: Meta-profiles of Cut&Tag signal +/- indicated minutes of triptolide treatment over active TSSs in A1-2 cells.

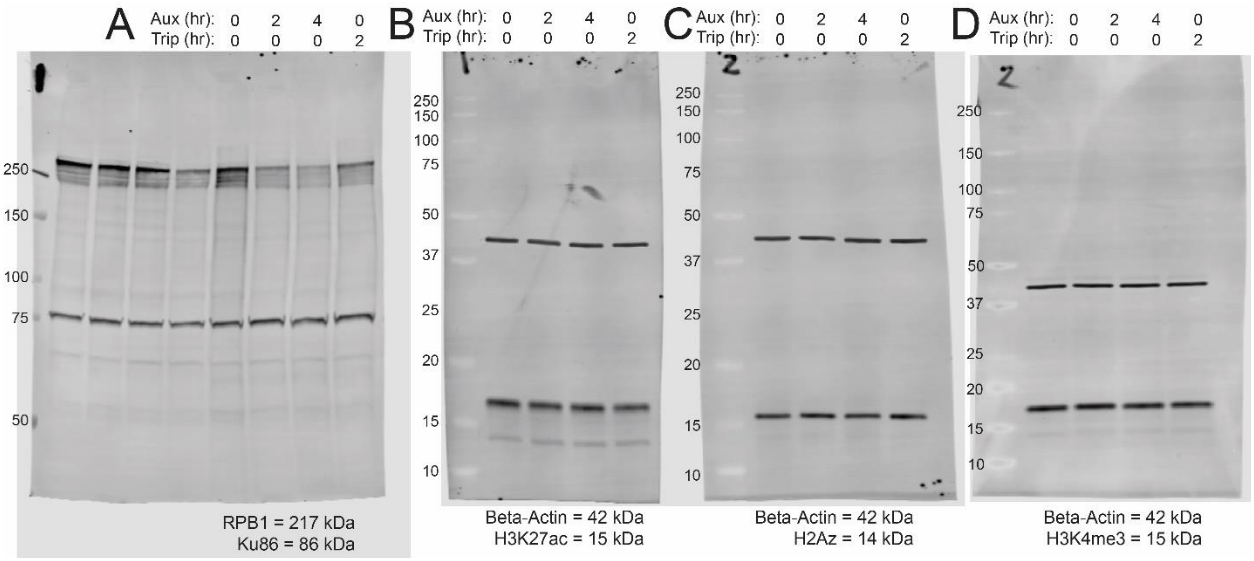

Figure S6: Uncropped western blot images corresponding to Figure 3E.

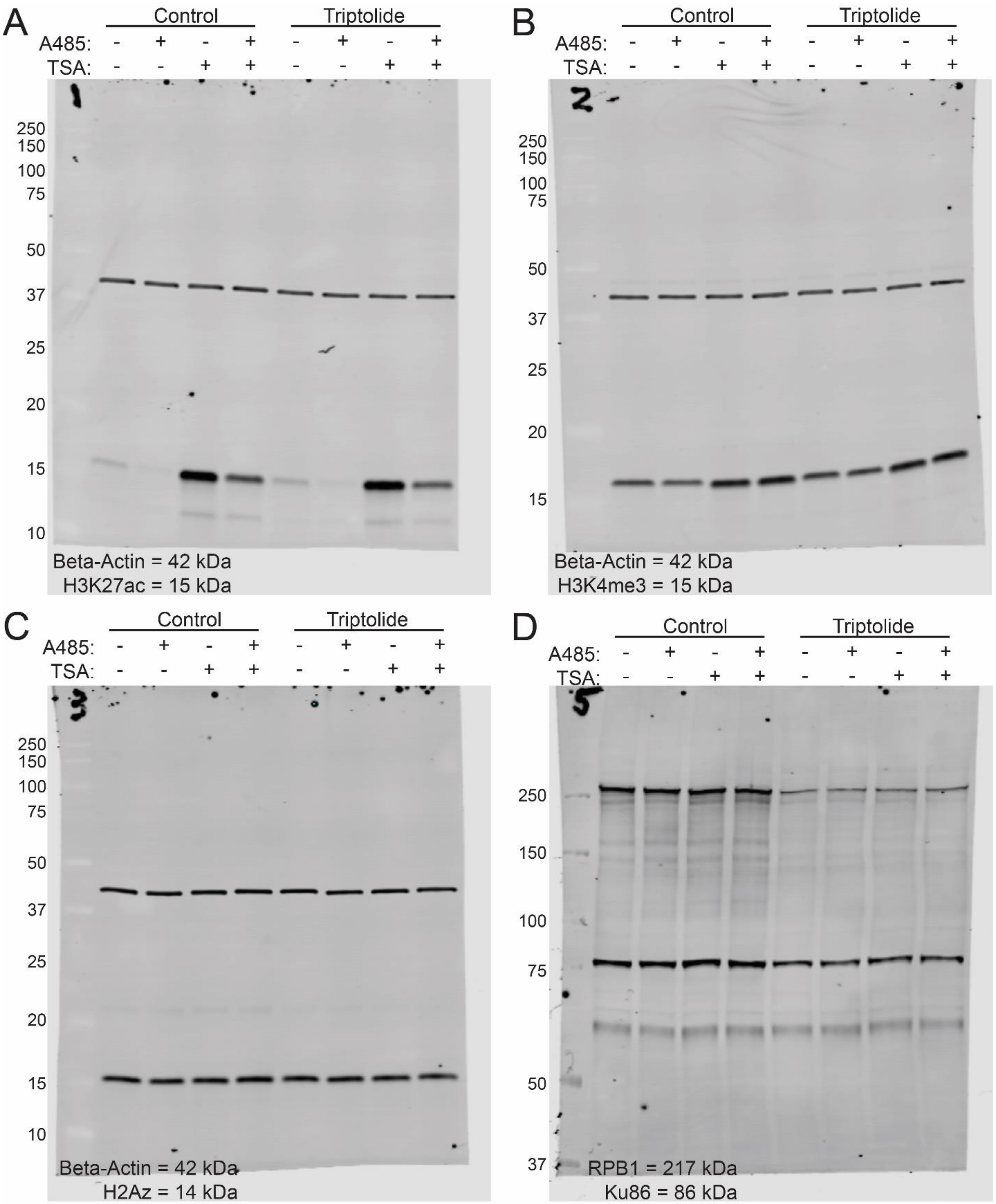

Figure S7: Uncropped western blot images corresponding to Figure 4A.

Table S1: Immunoblot antibodies

Target:	Cat. Num:	Vendor:	RRID:	Dilution:
RNAP2	39097	Active Motif	AB_2732926	0.2 ug/ml
Ku86-S10B1	sc-56136	Santa Cruz Biotechnology	AB_794204	0.2 ug/ml
H3K27ac	ab4729	Abcam	AB_2118291	0.5 ug/ul
β-Actin	A1978	Sigma-Aldrich	AB_476692	0.5 ug/ml
H2A.Z	39943	Active Motif	AB_2793401	0.5 ug/ml
H3K4me3	C15410003	Diagenode	AB_2924768	0.5 ug/ml
H3K18ac	C15410139	Diagenode	AB_2713907	0.5 ug/ml
H4K5ac	C15410025	Diagenode	AB_3661649	0.5 ug/ml

Table S2: CUT&TAG and ChIP-seq Antibodies

Target:	Cat. Num:	Vendor:	RRID:
H3K27ac	ab4729	Abcam	AB_2118291
H3K4me3	C15410003	Diagenode	AB_2924768
H3K9ac	C15410004	Diagenode	AB_2713905
H2A.Z	C15410201	Diagenode	AB_3661648
H3K27me3	C15410195	Diagenode	AB_2753161
H3K18ac	C15410139	Diagenode	AB_2713907
H3K14ac	ab52946	Abcam	AB_880442
RNAP2	39097	Active Motif	AB_2732926
H4K5ac	C15410025	Diagenode	AB_3661649
H4K12ac	C15410331	Diagenode	AB_3661650
H3K18ac	C15410139	Diagenode	AB_2713907
GR/NR3C1	NBP2-42221	Novus	AB_2894721
